# Combining field experiments and predictive models to assess potential for increased plant diversity to climate‐proof intensive agriculture

**DOI:** 10.1002/ece3.3028

**Published:** 2017-05-30

**Authors:** Norman W. H. Mason, David J. Palmer, Alvaro Romera, Deanne Waugh, Paul L. Mudge

**Affiliations:** ^1^ Landcare ResearchHamiltonNew Zealand; ^2^ DairyNZHamiltonNew Zealand

**Keywords:** biodiversity‐ecosystem function—BEF, chicory—*Chicorium intybus*, lucerne—*Medicago sativa*, plantain—*Plantago lanceolata*, ryegrass—*Lolium perenne*

## Abstract

Agricultural production systems face increasing threats from more frequent and extreme weather fluctuations associated with global climate change. While there is mounting evidence that increased plant community diversity can reduce the variability of ecosystem functions (such as primary productivity) in the face of environmental fluctuation, there has been little work testing whether this is true for intensively managed agricultural systems. Using statistical modeling techniques to fit environment–productivity relationships offers an efficient means of leveraging hard‐won experimental data to compare the potential variability of different mixtures across a wide range of environmental contexts. We used data from two multiyear field experiments to fit climate–soil–productivity models for two pasture mixtures under intensive grazing—one composed of two drought‐sensitive species (standard), and an eight‐species mixture including several drought‐resistant species (complex). We then used these models to undertake a scoping study estimating the mean and coefficient of variation (CV) of annual productivity for long‐term climate data covering all New Zealand on soils with low, medium, or high water‐holding capacity. Our results suggest that the complex mixture is likely to have consistently lower CV in productivity, irrespective of soil type or climate regime. Predicted differences in mean annual productivity between mixtures were strongly influenced by soil type and were closely linked to mean annual soil water availability across all soil types. Differences in the CV of productivity were only strongly related to interannual variance in water availability for the lowest water‐holding capacity soil. Our results show that there is considerable scope for mixtures including drought‐tolerant species to enhance certainty in intensive pastoral systems. This provides justification for investing resources in a large‐scale distributed experiment involving many sites under different environmental contexts to confirm these findings.

## 1 INTRODUCTION

Agricultural production systems are at increasing risk from severe weather events such as droughts. The development of agricultural practices that are resistant to climatic fluctuations will be a key component of adaptation to climate change (Howden et al., 2007; Lin, 2011). Over the past two decades, theoretical and field studies in ecology have demonstrated that increased species richness and functional diversity can enhance both the average rate and stability of ecosystem functions (such as primary production) in response to environmental fluctuations (e.g., Craven, Isbell, & Manning, 2016; Elmqvist et al., 2003; Isbell, Craven, & Connolly, 2015; Mori, Furukawa, & Sasaki, 2013; Tilman, 1996). However, there is very little work testing whether this is true for intensively managed agricultural systems. This study uses predictive models linking climatic conditions, soil properties, and biomass production to explore the scope for diversity to enhance both the mean and the stability of biomass production in intensive pastoral systems.

### 1.1 Could diversity enhance the mean and reduce the variability of production in intensively managed pastures?

Two contrasting hypotheses—niche complementarity (where temporal and spatial differences in resource use enhance function) and selection effects (where species identity has a strong influence on function)—have been proposed for explaining biodiversity‐ecosystem function (BEF) relationships (e.g., Loreau & Hector, 2001). Selection effects may be positive (inclusion of a competitive, high yielding species) or negative (reduction in abundance of high yielding species through competition Hector, Bazeley‐White, Loreau, Otway, & Schmid, 2002). Recent theoretical work has identified “response diversity”—interspecific differences in response to exogenous perturbations such as grazing or fluctuations in water availability—as a key factor in reducing variability in plant communities (Mori et al., 2013). Response diversity is required for “insurance effects” (Ives, Klug, & Gross, 2000) to occur. The insurance effect hypothesis and related concepts (Mcnaughton, 1977; Tilman, 1996; Tilman, Lehman, & Bristow, 1998) posit that variability in aggregate community properties, like productivity, will be reduced when decreases in production of some species in response to environmental fluctuation is compensated for through an increase in production by co‐occurring species that are favored, or less severely affected by that fluctuation. This is analogous to the niche complementarity hypothesis, but applied to the variability rather than to the mean of ecosystem function. Another possibility is that increasing diversity may reduce variability simply due to a greater chance of including species that are less sensitive to environmental fluctuations (e.g., fluctuations in water availability). This is essentially a positive selection effect, but for the variability, rather than for the mean, of ecosystem function.

### 1.2 The importance of climate–soil interactions for pastoral community variability

The scope for diversity to enhance stability is likely to depend on the intensity and frequency of climate fluctuations. For instance, we might expect differences in stability to be more evident in regions, or during periods, where agricultural systems experience climatic stress, such as drought (e.g., Tilman & Downing, 1994). Therefore, obtaining a more general picture of potential differences in stability between communities requires the assessment of stability under a range of climatic contexts. However, soil properties have a major influence on water stress experienced by plants, as soil water potential affects their ability to absorb water through their roots (Kramer & Boyer, 1995). In general, soils with larger water‐holding capacity will take longer to reach extreme soil water potential values during periods of drought (Heim, 2002). Thus, both soil properties and the intensity of climate fluctuations could influence stability directly while also affecting the potential for differences in species and functional diversity to enhance stability.

### 1.3 Using predictive models to explore the potential for diversity to enhance the mean and reduce the variability of ecosystem function

Properly comparing the mean and variability of ecosystem function for different plant communities requires long‐term (i.e., decadal) or large‐scale (i.e., >30 sites) experiments (Isbell et al., 2015; Tilman, 1996). This obviously demands a large amount of resources. Mathematical models have provided an initial step in confirming that diversity–stability relationships are theoretically possible in hypothetical communities (Ives, Gross, & Klug, 1999; Mori et al., 2013). Statistical or empirical models represent a next step where climate–productivity relationships observed in short‐to‐medium‐term experiments from one or several sites can be used to assess the variability of different communities for long‐term climate data over a large number of sites (Coomes et al., 2014). In this way, predictive models may provide some indication of the scope for alternative pasture mixtures to reduce variability, and thus aid in deciding whether or not resources should be devoted to long‐term and large‐scale experiments. In the past, this has proven to be a particularly useful approach for gauging the potential benefits of alternative forages for coping with climate change impacts (Chapman, Dassanayake, Hill, Cullen, & Lane, 2012).

### 1.4 Assessing pasture communities for reduced variability in productivity in new zealand

This study explores the scope for differences in the long‐term interannual variability in productivity of a two‐species and an eight‐species pasture mixture under intensive dairy farm management. The two‐species mixture consists of the standard perennial ryegrass (*Lolium perenne* L.)–white clover (*Trifolium repens* L.) mix employed by the majority of dairy farmers in New Zealand and is known to be very sensitive to soil water deficits (Nie, Chapman, Tharmaraj, & Clements, 2004). The eight‐species mix includes these two species and deep‐rooting forbs and legumes (drought avoiders). Swards containing these species have significantly higher productivity in dry conditions than the standard mixture (Woodward, Waugh, Roach, Fynn, & Phillips, 2013). By including a range of species that are less sensitive to drought than ryegrass and white clover, the eight‐species mixture provides greater response diversity with regard to drought stress, while also decreasing the overall drought sensitivity of pastures. It is therefore reasonable to expect that productivity in the eight‐species mixture ought to vary less in response to drought than the standard two‐species mixture, due either to niche complementarity or to positive selection effects on variability.

We begin by fitting statistical models linking climate variables, soil hydrological properties, and the productivity of individual harvests for the two‐species and eight‐species mixtures using data from 2‐ to 3‐year field experiments. We then use these models to make monthly predictions of productivity for long‐term (>30 years) climate data from the experimental study site on three soils with contrasting water‐holding capacity. We use these predictions to compare interannual coefficient of variation for productivity between mixtures and on different soils. Finally, these statistical models are used to assess the long‐term variability of mixtures for climate–soil combinations throughout New Zealand. Our approach allows us to test the following hypotheses relating to the mean and resistance of productivity:

(H1) There will be a general tendency for productivity in the eight‐species mixture to have lower variability than the two‐species mixture.
(H2) The eight‐species mixture will enhance mean productivity most on soils with low water‐holding capacity and in drier climates.
(H3) The eight‐species mixture will reduce variability in productivity most where climatic fluctuations are more severe and on soils with lower water‐holding capacity.

## 2 METHODS

### 2.1 Study site

The field experiments were located on Scott Farm near Hamilton in the North Island, New Zealand (37°46′16″S, 175°21′39″E). The mean annual temperature is 13.6°C, with a mean annual rainfall of 1224 mm. Winters are relatively mild (mean temperature in the coldest month is 4.2°C), and water deficit in summer and autumn is moderate to high (mean deficit—estimated using the Penman–Monteith equation for potential evapotranspiration—during summer and autumn was 71 ± 21 mm for the 3 years of the study). The soil at the experimental site is the Matangi silt loam (Typic Orthic Gley Soil; Hewitt, 1993). Typical annual production of grazeable herbage for pastures at Scott Farm is 15–22 t dry matter ha^−1^ year^−1^ with an average of 19 t ha^−1^ year^−1^ (Glassey, Roach, Lee, & Clark, 2013).

### 2.2 Small plot experiment

In March 2010, twelve different pasture mixtures were sown in 9 × 6 m plots (using a roller drill), following spraying with herbicide (glyphosate‐based) to kill the existing sward, mouldboard ploughing, and power harrowing. Three replicates of each mixture were sown in a randomized block design (see Mason et al., 2016; Fig. S1 for a schematic map of the experimental design). The two mixtures examined in this study were both based on perennial ryegrass (cv. “One50” inoculated with the AR1 endophyte). The “standard” mixture included white clover (cv. “Kopu II”) and perennial ryegrass (mixture code RGST). The diverse mixture included the white clover and two additional legume species (red clover, *Trifolium pratense* L. cv. “Colenso” and lucerne, *Medicago sativa* L. cv. “Torlesse”), two forb species (narrow‐leaved plantain, *Plantago lanceolata* L. cv. “Tonic” and chicory, *Chicorium intybus* L. cv. “Choice”), and two additional grass species (prairie grass, *Bromus willdenowii* Kunth. cv. “Atom” and timothy, *Phleum pratense* L. cv. “Charlton”). We chose these two mixtures as they contrasted strongly in diversity, differed significantly in productivity during the small plot experiment, displayed apparent differences in response of productivity to drought, and were both replicated in the “Large Plot Experiment” on the same experimental farm (thus providing a larger dataset for fitting complex models).

Management of the plots was designed to replicate, as much as possible, conventional dairy pasture management. Plots were grazed 10–12 times each year using 2–3 cows/plot (depending on estimated feed) for 2–3 hr. Cows were removed once residual feed was reduced to approximately 1,500–1,700 kg dry matter (DM) ha^−1^. The day before grazing, forage yield and botanical composition were estimated by harvesting a 0.85 × 5 m strip using a Jenquip™ harvester (Jenquip, Fielding, New Zealand). The cutting height of the harvester was set to 4 cm, which represents the optimum height of dairy cow grazing. Harvests were taken sequentially from the center of one of three 6 × 1 m evenly spaced strips, so that consecutive harvests were always taken from a different strip. The fresh weight of the harvested herbage was measured to the nearest 0.05 kg in the field. From this, a representative 1 kg sample was taken for dissection to individual species and dry weight measurement. Dry mass of the herbage sample and botanical components was determined by oven‐drying at 60°C for 24 hr.

### 2.3 Large plot experiment

Six replicates each of standard and complex perennial ryegrass‐based pastures were established in 0.5‐ha paddocks, with replicates randomly distributed among three blocks on a 3‐ha site at the same farm and on the same soil type as for the small plot trial (Woodward et al., 2013). Species composition and sowing rates were the same as for corresponding mixtures in the small plot trial, with the exception that timothy and red clover were not sown in the large plot trial complex mixtures. This is a minor difference as these species had universally low abundance (<5% of dry matter) within the small plot complex mixtures. Available herbage DM yield in each treatment paddock was estimated from cuts to grazing height (approximately 4–5 cm) before every grazing or silage cut. A Jenquip HT‐Kuma plot harvester was used to cut three 0.75 × 5 m strips (3.75 m^2^) in each paddock. From January 2013, a Haldrup F‐55 plot harvester (Haldrup Field Research Ltd. Germany) with cut width of 1.5 m (7.5 m^2^ cuts) was used. Herbage dry matter percentage and botanical composition were estimated following the same methods as for the small plot trial. Over 3 years, Woodward et al. (2013) reported virtually identical metabolizable energy (ME) for the standard and diverse pastures in the large plot experiment. Metabolizable energy (ME) was 11.7 MJ ME kg^−1^ DM for the standard mixture and 11.8 MJ ME kg^−1^ DM for the complex mixture. Thus, differences in dry matter production between mixtures are highly likely to reflect differences in energy intake by cows.

### 2.4 Soil moisture release curves

We obtained data on volumetric soil water content at different levels of soil water potential (ranging from −1,500 to −5 kPa) for three soil types—Matangi silt loam (on which both the small and large plot trials were located) and Horotiu and Waitoa silt loams (that also occur on Scott farm, but not under our experimental plots) and are common across the region in which our study site is situated. All three soils are formed on similar parent materials, alluvial rhyolitic sand, silt, and gravel overlaid with rhyolitic and andesitic ash. However, slight differences in the proportions of sand, silt, clay and gravels mean they have markedly different water‐holding capacities. The Matangi soil has the highest water‐holding capacity, followed by the Horotiu and then the Waitoa. Soil hydrological properties for the Matangi soil were derived from measurements made by Mudge et al. (2011), while data for the Horotiu and Waitoa soils were obtained from the National Soils Database (NSD, Landcare Research, 2015). Soil water content and water potential measurements were made for each “functional” horizon in the profile (to 1 m depth), with horizons being differentiated by changes in color and texture. Two samples were measured for each horizon. Further details on soil sampling and water content‐potential measurements are given in Gradwell (1972).

We used generalized linear models (GLM) with a Gaussian distribution and log link function to fit relationships between the negative reciprocal of soil water potential and soil water content. The relationship for the Matangi silt loam was used in fitting predictive models of productivity as this was the soil on which both the small and large plot trials occurred.

### 2.5 Modeling climate and soil water content effects on productivity

Daily climate data for fitting productivity models were taken from the virtual climate station network (VCSN, NIWA, 2015) station closest to our study site. The VCSN is an interpolated climate surface with “stations” located on a 5‐km grid. We chose to use VCSN data in fitting BRT models to facilitate the use of these models to make predictions for the entire VCSN network (see section “National predictions for different soil‐climate combinations” below). We used the WATYIELD water balance model (Fahey et al., 2010) to estimate soil water content to 1 m for each of our three soils based on rainfall and potential evapotranspiration (PET) data obtained from the VCSN and soil hydrological properties obtained from Mudge et al. (2011) and the NSD (Landcare Research, 2015). Soil water content–potential relationships were used to estimate daily soil water potential, for inclusion in productivity models.

We used boosted regression trees (BRT, Elith, Leathwick, & Hastie, 2008) to model mean daily pasture dry matter production (kg ha^−1^ day^−1^) of each harvest for the standard and complex mixtures. BRT is a machine‐learning technique where multiple regression trees (models that relate a response to its predictors by recursive binary splits) are fitted in a forward, stagewise fashion to produce an additive ensemble model. Average daily values during the intervals between harvests for the climate variables and soil water potential were used as predictors. BRT models were fitted using a learning rate of 0.001 and a tree complexity of 3 (meaning the interactions between as many as three variables could be modeled). Model goodness of fit was assessed by cross‐validation, where the data are separated into training and evaluation subsets. We used block as a “fold vector,” meaning that in cross‐validation all the data from a given block were excluded from the training data set, with goodness of fit being assessed by predicting on to the excluded block. This approach incorporates the repeated‐measures structure of the productivity data in assessing goodness of fit.

A model simplification procedure—implemented by the gbm.simplify R function (Elith et al., 2008)—was used to reduce the number of variables. Climate variables used in the final models were potential evapotranspiration (PET, mm), solar radiation (MJ/m^2^), and minimum daily temperature (*T*_min_, °C). We deliberately excluded variables relating to moisture availability to ensure that this was expressed uniquely via soil water potential and thus enable more accurate extrapolation to soils other than the one on which the two experiments were conducted. We chose not to include soil fertility in the predictive models as the experimental data on which the models are based were obtained from pastures experiencing high rates of nutrient inputs. Nitrogen (N) fertilizer (urea) was applied strategically postharvest, targeting 200 kg N ha^−1^ year^−1^. Maintenance fertilizer (P = 35 kg ha^−1^ year^−1^; K = 117 kg ha^−1^ year^−1^; S = 50 kg ha^−1^ year^−1^ was applied in autumn of each year. Thus, productivity in our experimental pastures was much less likely to be limited by nutrient availability than by temperature and water availability. The experimental period incorporates a severe drought (in the summer and autumn of the final year), so that the models are based on harvest data spanning a very wide range of soil moisture availability.

We began by fitting models on the small plot data for each mixture and then evaluating them on the large plot data for the corresponding mixture. There was very little difference in the goodness of fit obtained in cross‐validation (i.e., prediction on to small plot data excluded from the training dataset) and that obtained when predicting on to the large plot data (Fig. S1). This suggests that productivity in the small and large plots responded to the predictors in a similar way. Based on this, we decided to group the small and large plot data together in fitting productivity models. All BRT modeling was performed in R using code provided in the supplementary material of Elith et al. (2008).

### 2.6 Predicting productivity using long‐term climate data for different soils at the field trial site

We used the BRT models fitted using the small and large plot productivity data to model productivity on long‐term (1980–2013) daily climate data from the same VCSN station we used to fit the models for the Matangi soil. These climate data were used to estimate soil water content for all three soils via the WATYIELD water balance model. Fitted soil water content–soil water potential relationships were then used to estimate daily soil water potential. Productivity was modeled on a monthly basis, with annual productivity being estimated as the mean (weighted by days in each month) productivity taken across months. We used permutation tests to test for significant differences in the mean and variance of annual productivity for each mixture on the three different soils. The permutations randomly allocated annual productivity values for each soil to either mixture, with mean and variance being calculated for each permutation. This randomization strategy is suitable for a single‐factor significance test (Anderson & Ter Braak, 2003). We also summarized long‐term predictions for the experimental site seasonally, with seasons defined as spring (1 September–30 November); summer (1 December–28/29 February); autumn (1 March–31 May); and winter (1 June–31 August).

### 2.7 National predictions for different soil–climate combinations

We then conducted a scoping study to determine the potential differences in pasture production between the standard and diverse mixes if results were extrapolated to a broader range of soil–climate combinations. For this scoping study, we wanted to ensure we had soils which represented the range of water‐holding characteristics of New Zealand soils. Therefore, we first obtained hydrological data from the NSD (Landcare Research, 2015) for all soil profiles (290) that had been sampled to >0.9 m (excluding organic soils), and calculated total profile available water (TAW) and readily available water (RAW) to 1 m depth. The distribution of TAW revealed that the Matangi soil was near the 90th percentile (257 mm) and the Horotiu soil the 50th percentile (182 mm). We therefore selected a soil on the 10th percentile (112 mm) for TAW (Wingate), so the range of soils in New Zealand was represented. Our intention was to illustrate how interactions between soil water‐holding capacity and climate might influence the relative performance of mixtures, rather than provide a representation of agricultural soils across New Zealand.

**Figure 1:**
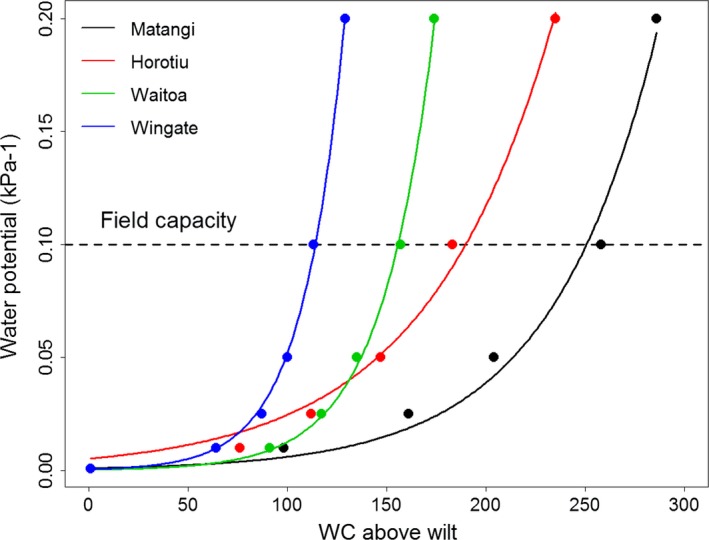
Relationships between water content (mm above wilting point) and soil water potential (expressed as −1/kPa) for the three soils at Scott Farm (Matangi, Horotiu, and Waitoa) and an additional soil representing a nationally very low TAWC (Wingate). Field capacity (−10 kPA) is shown as a dashed line. Fitted data were obtained using generalized linear models with a Gaussian distribution and log link function. A decrease in soil water potential makes it more difficult for plant roots to absorb water. The differences in water content at field capacity mean the soils differ in the amount of water loss required to achieve low water potential values, with the Matangi soil taking longer to reach low water potential during dry periods, and the Wingate soil reaching low water potential very rapidly

We then predicted productivity values for the standard and diverse pasture mixtures for the three representative soils onto long‐term climate data for all VCSN stations in New Zealand (over 11,000 in total). Thus for each VCSN station, we had predictions of pasture production for three soils with quite different water content to water potential relationships (Figure 1).

For each station, we calculated the difference in the mean and variance of annual productivity between mixtures and applied the permutation tests outlined above to test for significant differences in mean and variance for each soil in each virtual climate station. In comparing the performance of the two pasture mixtures, we focussed on VCSN stations in areas suitable for intensive pastoral farming (Newsome, 1992).

### 2.8 Recreating observed patterns without interspecific interactions

We devised a very simple model documenting the response of productivity to a hypothetical soil water availability gradient for two‐species mixtures containing either two drought‐sensitive species or one drought‐sensitive and one drought‐tolerant species. Both species in either mixture were assumed to have equal abundance, reflecting a scenario where the relative abundances of each species are not influenced by competition. We calculated mean annual productivity and the coefficient of variance for productivity for each mixture in random draws of 40 “years” of soil water availability data (1,000 in total, using a uniform probability distribution and with replacement). Difference in mean and coefficient of variation of annual productivity between the two mixtures was recorded for each random draw. Annotated R code and input data for this model are provided in the Appendices S1 and S2.

## 3 RESULTS

### 3.1 Soil moisture release curves

The four soil types we examined differed markedly in their hydrological properties. The Matangi soil had a very large water‐holding capacity (i.e., water content at field capacity (−10 kPa) minus that at wilting point (−1,500 kPa), with that of Horotiu being intermediate, Waitoa low and Wingate very low (Figure 1). Consequently, soil water potential for the Matangi changed very slowly with changes in water content relative to the Wingate soil, with the Horotiu soil having an intermediate rate of change.

### 3.2 Boosted regression tree models of productivity for pasture mixtures

**Figure 2:**
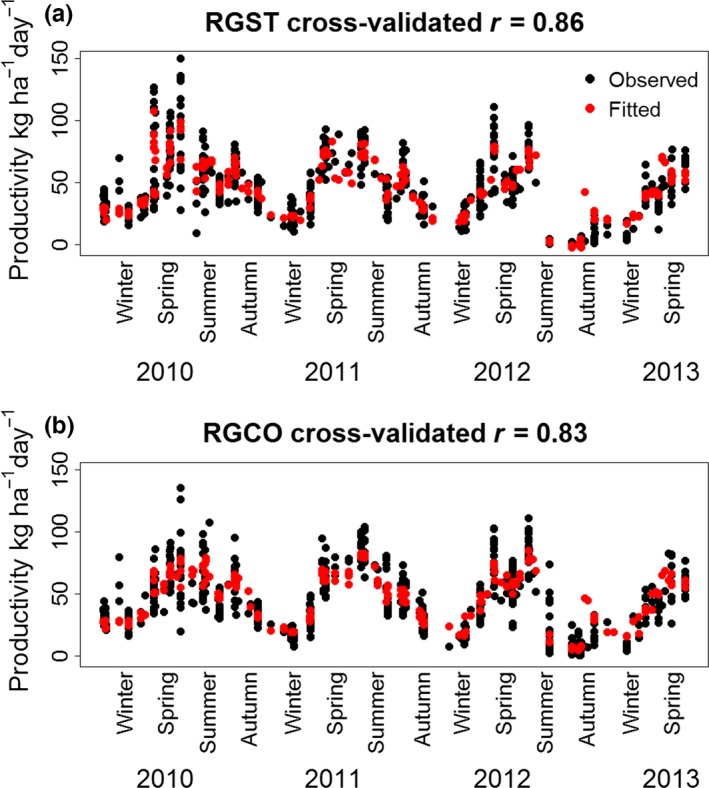
Observed and fitted productivity of (a) the standard (RGST) and (b) complex (RGCO) mixtures for individual harvests in the small and large plot trials over 3.5 years. The fitted values presented for each harvest are derived from boosted regression tree (BRT) models excluding the harvest in question (see Methods for details on cross‐validation)

Reasonably accurate BRT models were obtained for both mixtures, with cross‐validated correlations between observed and fitted values of *r* = 0.86 and *r* = 0.83 (or 74% and 68% of variation explained) for the standard and complex mixtures respectively (Figure 2). Soil water potential and solar radiation made the largest contribution to the BRT model for the standard mixture (i.e., were used most frequently to divide the dataset—form branches—in regression trees, Table 1), while PET made the largest contribution to the complex mixture productivity model, followed by soil water potential.

**Table 1:** Contribution of predictor variables in productivity models for standard and complex mixtures, expressed as the percentage of times a variable is used to form branches in regression trees

Predictor	Standard	Complex
Water potential	37	33
PET	24	41
Solar radiation	34	20
Tmin	5	5

PET, potential evapotranspiration; *T*_min_, minimum daily temperature.

**Figure 3:**
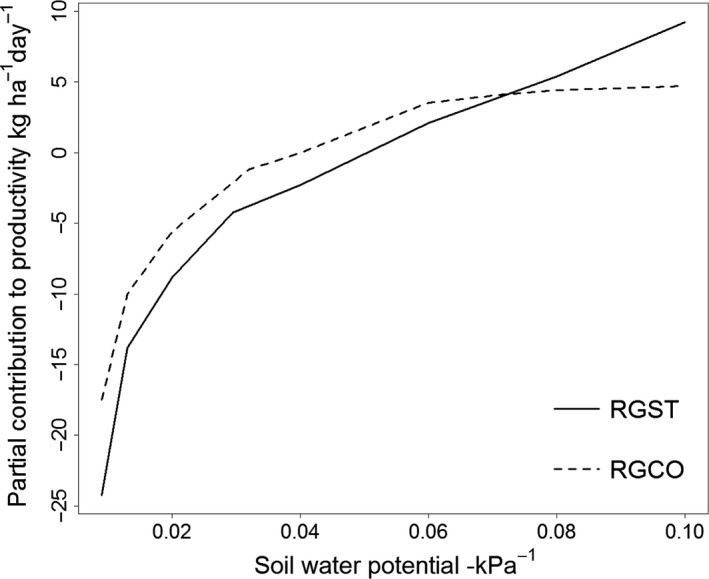
Smoothed partial response curves (i.e., with mean values assigned to all other predictors) of daily dry matter productivity against water potential (−1/kPa) for standard (RGST, solid line) and complex (RGCO, dashed line) mixtures. Note that the productivity for the ryegrass standard declines as soon as water potential is below that at field capacity (0.1 −kPa^−1^), while productivity of the complex mixture does not decline noticeably until comparatively low water potentials are reached

The two mixtures differed markedly in their partial response (i.e., with all other predictors held at their mean values) to soil water potential. Productivity for the standard mixture begins to decline as soon as water potential falls below that at field capacity (i.e., water potential <0.1), while productivity for the complex mix does not decline markedly until water potential is well below potential at field capacity (i.e., water potential <0.05, Figure 3). Further, partial contribution values for the standard mixture span a larger range (−24 to 10 kg ha^−1^ day^−1^) than for the complex mixture (−18 to 4 kg ha^−1^ day^−1^), indicating that variation in soil water potential exerted a greater influence on variation in productivity for the standard mixture. In other words, the productivity of the standard mixture was more sensitive to declines in soil water availability than the complex mixture. Partial response curves for all predictors in the productivity models for standard and complex mixtures are given in the Figs. S2 , S3.

### 3.3 Using productivity models to compare long‐term performance of mixtures on different soils

#### 3.3.1 Experimental study site

Predicted long‐term mean annual productivity using climate data from the closest virtual climate station did not differ significantly between mixtures for any of the soil types occurring at the study site (Fig. S4A). However, variance in annual productivity was significantly higher for the standard mixture across all soil types (Fig. S4B). This suggests that productivity in the complex mixture was more resistant to climatic fluctuations between years than in standard mixture.

Looking at seasons separately, the standard mixture tended to have higher productivity in the winter and spring, particularly on Matangi and Horotiu soils (Fig. S5). By contrast, the complex mixture tended to have higher productivity in the summer and autumn, particularly on the Waitoa soil. This variation in the relative productivity of mixtures with soil type is consistent with the differences in partial response to soil water potential. The standard mixture, which is sensitive to variation in water potential, tends to perform best on the soil with the largest water‐holding capacity (Matangi), and worst on the soil with the smallest water‐holding capacity (Waitoa). The one exception to this was in autumn, when the relative performance of the standard mixture was best on the Horotiu soil. Closer inspection of seasonal variation in soil water potential suggests this occurred because the Horotiu soil requires a smaller increase in water content relative to the Matangi soil to achieve the same level of increase in water potential, meaning that its water potential increases more rapidly after summer dry periods.

#### 3.3.2 All New Zealand

At the national scale, the relative performance of the standard and complex mixtures was evenly balanced for soils with high (Matangi) and moderate (Horotiu) water‐holding capacity across VCSN sites (Table 2). The median value for differences in mean annual production in both cases was close to zero, while the standard had significantly greater mean annual productivity than the complex on a slightly smaller percentage of sites than *vice versa* (27% vs. 30% for Matangi soils and 21% vs. 35% for Horotiu soils, Table 2). By contrast, for the low water‐holding capacity Wingate soil the complex mixture yielded, on average, at least 900 kg ha^−1^ year^−1^ more than the standard mixture across the majority of VCSN sites (median difference = 948), and had a significantly higher mean annual productivity for a large (62%) percentage of sites. Thus, for soils with moderate–high water‐holding capacity the relative performance of either mixture is likely to be very dependent on climatic conditions, while on soils with low water‐holding capacity the complex mixture consistently performs better across all climate types examined.

**Table 2:** Median and 90% confidence interval bounds (5th and 95th percentile) for differences between the standard (RGST) and complex (RGCO) mixtures in mean and coefficient of variation of annual productivity for soils with high medium and low water‐holding capacity (Matangi, Horotiu, and Wingate, respectively) across virtual climate stations in areas suitable for intensive agriculture and percentage of stations where differences between mixtures were significant (*p* < .05)

	Percentile (kg ha^−1^ year^−1^)			Percent significant differences		
	5th	Median	95th		RGST > RGCO	RGST < RGCO
*Mean*						
Matangi	−1,239	40	1,781	Matangi	27	30
Horotiu	−637	42	870	Horotiu	21	35
Wingate	81	948	2,012	Wingate	1	62
*Coefficient of variation*						
Matangi	−1.30	−0.32	−0.09	Matangi	73	0
Horotiu	−0.21	−0.11	−0.05	Horotiu	87	3
Wingate	−1.19	−0.51	−0.15	Wingate	45	0

Negative values for percentiles indicate that RGST is greater than RGCO.

The coefficient of variation in annual productivity was consistently lower for the complex than the standard mixture across VCSN sites, irrespective of soil type (Table 2). The soils did differ markedly in the percentage of sites where variance differed significantly between mixtures (72% for Matangi, 87% for Horotiu and 45% for Wingate, Table 2). The lower percentage of significant results for the Wingate soils arose because both mixtures were often prone to occasional years of extreme high or low production. These results strongly suggest that the productivity of the complex mixture will be less sensitive to interannual climatic variation across a wide range of climatic regimes and soil types.

#### 3.3.3 Effect of soil water potential on relative performance of mixtures

**Figure 4:**
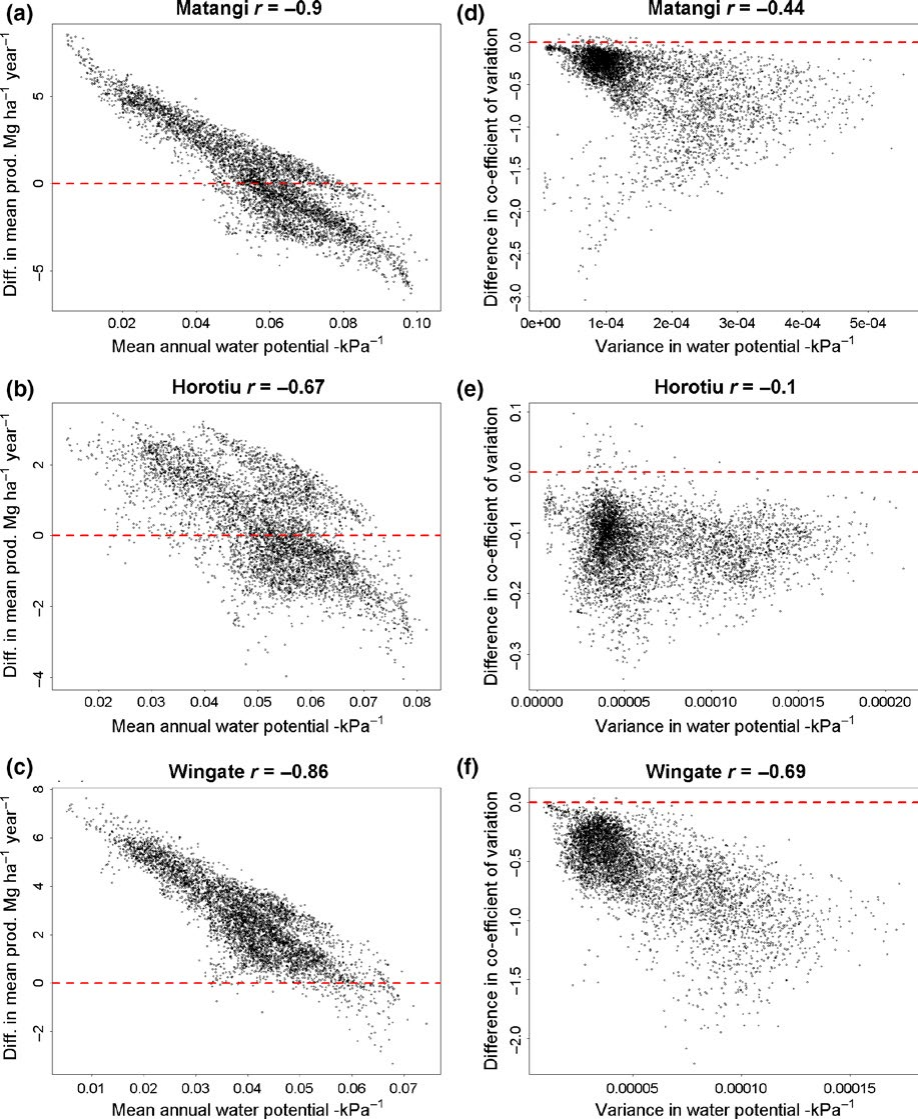
Differences in mean annual dry matter yield (a–c) and the coefficient of variation for annual productivity (d–f) between the standard and complex mixtures against (respectively) the mean and variance of annual soil water potential for soils with high, medium, and low water‐holding capacity (Matangi, Horotiu, and Wingate, respectively) across virtual climate stations in areas suitable for intensive agriculture. Negative values indicate the standard was greater than the complex. Low values for soil water potential indicate soil water is less available to plants

Differences between mixtures in long‐term mean annual productivity across virtual climate stations were very strongly negatively related to mean annual soil water potential for the Matangi and Wingate soil (*r* = −0.9 and −0.86 respectively, Figure 4) and moderately strongly negatively related for the Horotiu soil (*r* = −0.67). This shows that the mean productivity of the complex relative to the standard tended to be greater in areas experiencing lower soil water availability. This in turn suggests the advantage of sowing the complex mixture will be greatest in dry climates, irrespective of soil water‐holding capacity.

Differences in the coefficient of variation of annual productivity (CV) were negatively correlated with variance in annual water potential for the Matangi and Horotiu soils (*r* = −0.44 and −0.69, respectively, Figure 4), with the standard mixture tending to have much higher CV when variance in water potential was higher. The corresponding relationship was quite weak for the Horotiu soil (*r* = −0.1), though still in the same direction.

## 4 DISCUSSION

Our study illustrates the potential power of combining field experiments with predictive models incorporating climate–soil interactions to gauge the potential benefit of increased diversity for intensive agricultural production over large spatial scales. We were able to show that the more diverse mixture is likely to have consistently lower interannual variation in productivity, irrespective of soil type or climate regime. This in turn strongly suggests that the more diverse mixture will generally reduce the sensitivity of productivity to interannual climatic fluctuations across the full range of climate–soil combinations encountered in New Zealand's agricultural landscapes. The relative difference in variation between mixtures was related to climate for the high and low water‐holding capacity soils, being greatest in areas with high variance in annual soil water potential. This relationship was much weaker for the moderate water‐holding capacity soil.

We also show that the more diverse mixture enhanced mean annual productivity, but this was strongly dependent on both climate and soil type. The relative performance of mixtures was quite evenly balanced at the national scale for the soils with high and moderate water‐holding capacity. By contrast, there was a consistent pattern for the more diverse mixture to outperform the standard mixture on the soil with low water‐holding capacity. Further, for all three soils the enhancing effect of the diverse mixture on mean annual productivity was greatest in drier climates (where mean annual water potential was lowest). This suggests that the reduced sensitivity to interannual climatic fluctuations gained through sowing the complex mixture may come at the cost of lower mean annual productivity where soils with high water‐holding capacity coincide with wetter climatic conditions, while in drier areas and on soils with low water‐holding capacity, the complex mixture will enhance productivity. Below we explore the implications of our results for future proofing agriculture against climate change in an increasingly resource‐limited world. We also discuss the possible ecological mechanisms driving our results and how future studies could be designed to predict the relative influence of competing mechanisms over large spatial scales.

### 4.1 Could diversity increase agricultural certainty in increasingly uncertain climates

Our findings show that more diverse mixtures could have considerable benefits for both the mean and stability of production in intensive agricultural systems in a future where climatic fluctuations, particularly in temperature and rainfall, are expected to be more intense and more frequent (Allan & Soden, 2008; Fischer & Knutti, 2015). With specific reference to New Zealand, many areas are expected to experience drought more frequently under climate change (Clark, Mullan, & Porteous, 2011), so the more diverse mixture, by providing greater mean and stability in productivity of pastures under dry climates, is likely to be increasingly advantageous in the future. Another advantage of the more diverse mixture could be lower irrigation requirements for the maintenance of productivity, which will become an increasingly important consideration as economic development and climate change place intensifying demands on freshwater resources (WWAP, 2012).

However, our findings only represent a first step in assessing the potential benefit of the more diverse mixtures. First, our results only give an idea of the potential benefit of more diverse pastures for productivity. To confirm our findings would require a large‐scale study involving many sites under different environmental contexts. The power of such distributed trials is becoming increasingly apparent (Craven et al., 2016; Isbell et al., 2015; Finn, Kirwan, & Connolly, 2013). The potential benefit of sowing more diverse pastures suggests there may be merit in investing the resources required for such a trial comparing diverse sward performance to that of the standard ryegrass–white clover mixture, particularly in dry climates and on soils with low–moderate water‐holding capacity. Further, we have not considered the effect of soil fertility as the intensively managed pastures in which our field experiments were conducted are typified by high rates of nutrient inputs, meaning that productivity is unlikely to be limited by nutrient availability. However, the best available evidence suggests that plant diversity effects on productivity are not influenced by soil fertility (Craven et al., 2016), so that it is unlikely variation in soil fertility would affect our main conclusions.

Another consideration is the difficulty in maintaining diversity in pasture mixtures. A previous study of intensively managed grazed pastures showed that the abundance of chicory, plantain, and lucerne had declined markedly after 3 years (Mason et al., 2016). The requirements of intensive pastoral agriculture—the ability to produce large amounts of high‐quality herbage while tolerating frequent defoliation and trampling—place severe constraints on the pool of viable species (Finn et al., 2013). It is likely that changes in management practices will be required to maintain diversity, as well as the development of new cultivars for drought‐resistant species (Woodward et al., 2013). Finally, the ultimate criterion for any change in management is benefit to the farm as a whole, including effect on profitability, environmental impacts, complexity of the change, and how it fits within the current regime (Pannell et al., 2006). Assessing this will require, initially, integration of productivity models with whole‐farm economic models (Beukes et al., 2008) and then farm‐scale trials that monitor production and profitability when the more diverse mixture is sown.

### 4.2 Diversity and the mean and variability of annual productivity

Our findings agree with available evidence for forests that the enhancing effect of diversity on mean ecosystem function is greatest under adverse environmental conditions (e.g., Jucker et al., 2016; Paquette & Messier, 2011), but contrast recent evidence from grassland experiments showing that diversity‐ecosystem function relationships are robust against both nutrient addition and drought (Craven et al., 2016; Hofer et al., 2016). The forbs chicory (*Chicorium intybus*) and plantain (*Plantago lanceolata*) and the legume lucerne (*Medicago sativa*), when grown as monocultures, have all been shown to have greater drought resistance in agricultural production systems than perennial ryegrass (*Lolium perenne*)–white clover (*Trifolium repens*) mixtures, or to enhance resistance of productivity to drought when added to ryegrass–white clover mixtures (Mills, Smith, Lucas, & Moot, 2008; Rollo et al., 1998; Skinner, 2008; Stewart, 1996). Therefore, it is unsurprising that the more diverse mixture enhanced mean annual productivity most in drier climates (Figure 4). The more diverse mixture was also less sensitive to variation in soil water availability than the standard ryegrass–clover mixture (see Figure 3). So it is also unsurprising that the more diverse mixture generally had greater resistance (lower variance) in productivity than the standard mixture.

With our study design, it is difficult to make definitive statements about ecological mechanisms behind differences in the mean and variance of productivity between our mixtures. The instances where the standard mixture performs better than the more diverse mixture are probably due to negative selection effects (Loreau & Hector, 2001), with reduction in the relative abundance of ryegrass and white clover (due to competition with additional species) in the more diverse mixture limiting its ability to increase production in response to greater soil water availability. Instances where the more diverse mixture performs better could be due either to niche complementarity or positive selection effects. A previous study showed that adding the winter dormant forbs chicory and plantain to ryegrass–white clover mixtures enhances mean productivity due to their phenological (seasonal) complementarity with winter‐active ryegrass (Mason et al., 2016), suggesting there may be some potential for niche complementarity to enhance productivity even in intensively managed agricultural ecosystems. Other studies have emphasized the potential for increased diversity to enhance productivity through complementarity in nitrogen resource use between legumes and non‐N‐fixers (e.g., Nyfeler, Huguenin‐Elie, Suter, Frossard, & Lüscher, 2011). In our field experiments, the two mixtures had similar proportions of biomass contributed by legumes, except in the summer and autumn of the final year (Fig. S6A), when a severe drought occurred. However, this was largely due to an increase in the abundance of drought‐tolerant Lucerne (Fig. S6B) and thus reinforces the interpretation that including drought‐resistant species enhanced the resilience of the complex mixture. Ultimately, distinguishing between these hypotheses requires a mixture‐monoculture experimental design, where all species included in mixtures are also grown as monocultures (Loreau & Hector, 2001).

It is also difficult to demonstrate definitively from our results whether the reduced variability of the diverse mixture is due to its greater response diversity (Mori et al., 2013), or simply due to the inclusion of drought‐resistant species in the more diverse mix.

This dichotomy is similar to the contrast between selection effects and niche complementarity for mean annual productivity. Inspection of the observed productivity for individual harvests from the small and large plot trials (Figure 2) shows that the standard mixture had higher peaks (particularly in spring) and lower troughs (particularly during dry periods in summer and autumn). Thus, both the positive and negative fluctuations in productivity were less marked in the more diverse mixture, which is consistent with the generally lower variation observed for the diverse mixture. This is also consistent with available evidence suggesting that diversity enhances resistance of productivity to both increases and decreases in water availability (Isbell et al., 2015). However, it does not reveal whether selection effects or niche complementarity play a greater role in the enhanced resistance of the more diverse mixture. Distinguishing between the two would also require a mixture‐monoculture experimental design.

### 4.3 Combining monoculture‐mixture designs and predictive models to understand spatial variation in diversity effects on ecosystem function

**Figure 5:**
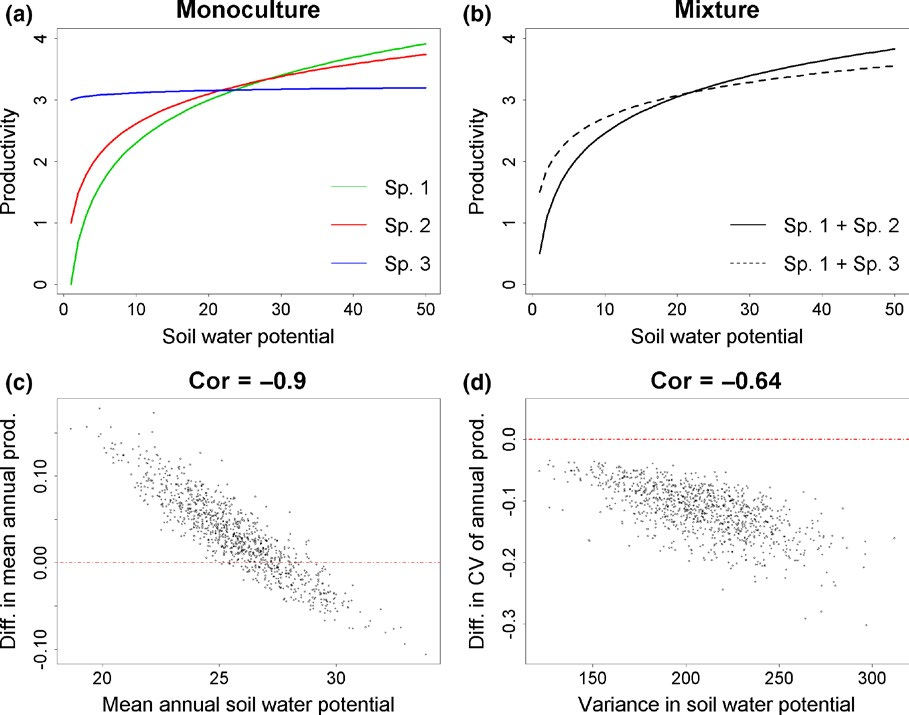
Response of productivity to variation in soil water potential for monocultures of three hypothetical species (a); Response of productivity for two‐species mixtures including either the two drought‐sensitive species (Sp. 1 and Sp. 2), or the most drought‐sensitive species and the most drought‐resistant species (Sp. 1 and Sp. 3, respectively), assuming species have equal abundance in mixtures (b); difference in mean and coefficient of variation of annual productivity between the two mixtures (c, d) against mean and variance of annual water potential, respectively. Each data point in (c) and (d) summarizes differences between mixtures in random draws of 40 “years” (1,000 in total, using a uniform probability distribution and with replacement) from the soil water potential–productivity relationships in (b)

It is possible to recreate patterns similar to those observed in Figure 4 using a very simple model comparing productivity in two‐species mixtures composed either of two drought‐sensitive or a drought‐sensitive and drought‐resistant species (Figure 5, see supplementary files for R script and input data). Basically, the difference in mean annual productivity between mixtures with or without a drought‐resistant species is strongly correlated with mean annual soil water potential, while the difference in variance between mixtures is not. This model does not include any species interactions, with each species assigned equal abundance in mixtures irrespective of environmental conditions, and thus is consistent with the findings of Ives and Carpenter (2007) that interspecific competition is not required for diversity‐stability relationships to occur. Further, it is obvious from this example that similar results could be obtained comparing monocultures of drought‐resistant species to mixtures dominated by drought‐sensitive species. This suggests that the simple inclusion of drought‐resistant species, rather than greater response diversity in the more diverse mixture might be sufficient to explain our results. In other words, the strong relationship between differences in mean annual productivity and soil water potential might largely be due to a shift from negative to positive selection effects in the more diverse mixture with decreasing water potential. Similarly, the greater resistance of productivity in the diverse mixture might be largely explained by the inclusion of species that are less responsive to variation in water potential, being largely independent of response diversity.

This simple model example and our results also serve to illustrate the potential power of combining a mixture‐monoculture design with a predictive modeling approach to identify where niche complementarity and positive or negative selection effects are likely to be strongest. Intensively managed pastures have the advantage of very frequent harvests (up to 14 per year), meaning that a large amount of data can be obtained from an experiment lasting several years. This might make it feasible to use complex data mining techniques like boosted regression trees (Elith et al., 2008) to model climate–productivity relationships for monocultures and mixtures alike. Such models could be used to make large‐scale spatial predictions for monocultures and mixtures. This in turn would allow large‐scale estimation of potential over‐yielding (a key criterion for differentiating between mechanisms behind BEF relationships Loreau & Hector, 2001), as well as estimation of the relative influence of niche complementarity and selection effects in diversity–resistance relationships under a broad range of environmental contexts.

## 5 CONCLUSIONS

Combining predictive models and field experiments could be a powerful approach for extracting the maximum value out of hard‐won experimental data. We were able to employ this approach in demonstrating that while the relative productivity of a more diverse pasture mixture is highly dependent on climatic conditions and soil water‐holding capacity, the more diverse mixture had greater resistance in productivity irrespective of soil type, or the mean and variance of soil water availability. We were also able to show that the more diverse mixture had greater resistance in productivity irrespective of soil type, or the mean and variance of soil water availability. However, a large‐scale distributed experiment involving many sites under different environmental contexts would be required to confirm these findings.

Our findings illustrate how an appropriate modeling approach makes it possible to use small‐scale, medium‐term experiments to gauge the potential long‐term benefits of a more diverse mixture for intensive pastoral agriculture at the national scale. Such an approach, when combined with mixture‐monoculture experiments, could open up exciting new possibilities for mapping the relative contribution of competing ecological mechanisms in biodiversity‐ecosystem function relationships. This could ultimately provide invaluable insights on how to manage intensive agricultural systems to maximize the benefits of increased diversity.

## CONFLICT OF INTEREST

None declared.
