# Supplementary material for: Combining field experiments and predictive models to assess potential for increased plant diversity to climate‐proof intensive agriculture
Source: Ecol Evol. 2017 May 30;7(13):4907–18. doi: 10.1002/ece3.3028 (PMC5496536; doi:10.1002/ece3.3028)
Supplement: Supplementary file 1 [file ECE3-7-4907-s001.docx]

Figure S1: Observed productivity in large plot harvests against predicted values from BRT models fitted on small plot harvest data for the standard (RGST) and complex (RGCO) mixtures. CVcor is the cross-validated correlation for BRT models fitted on small plot data while Validation cor is the correlation between predicted values from small plot BRT models and observed values from large plots.


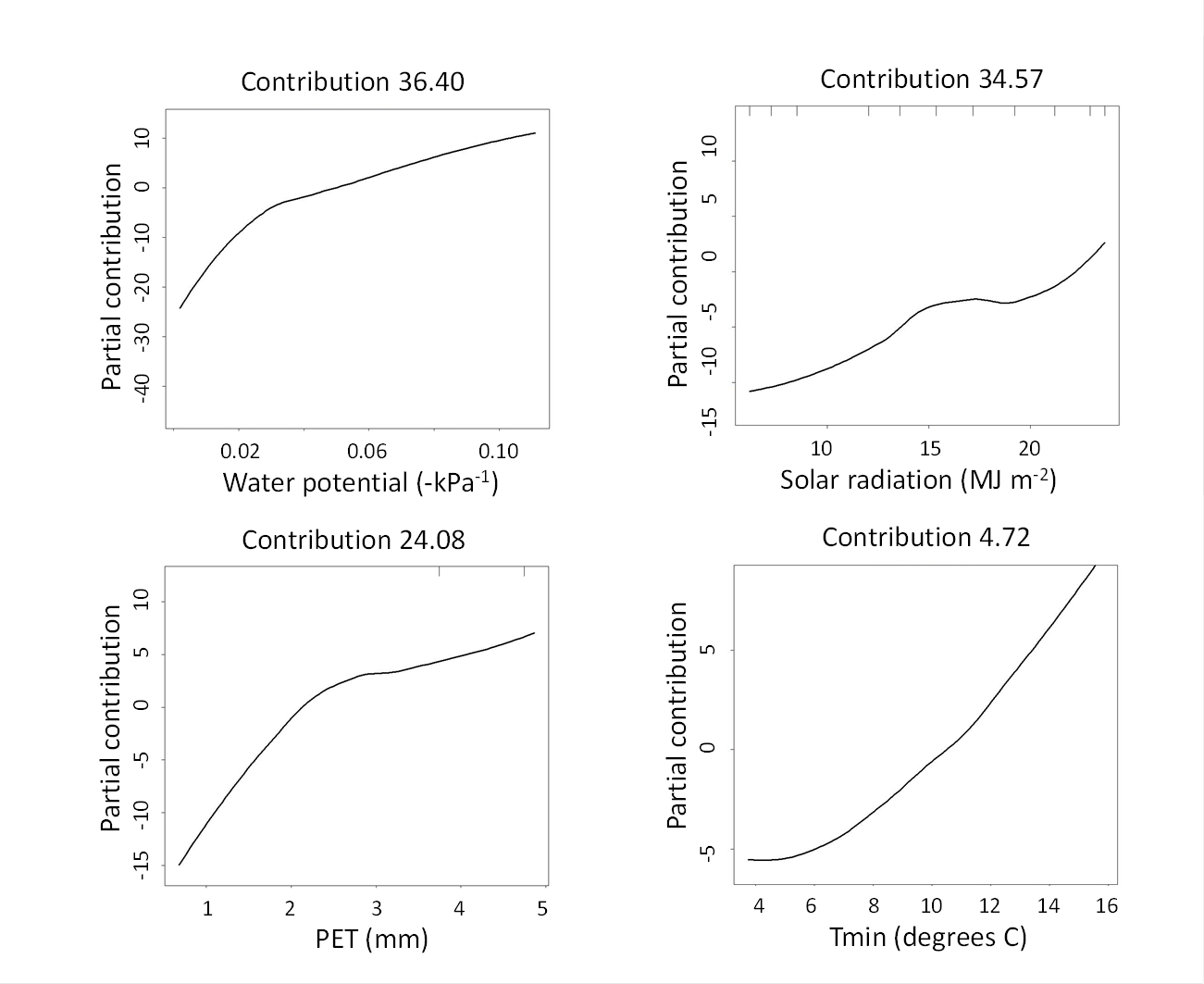


Figure S2: Partial response of predictors to productivity in the BRT model for the standard mixture (RGST). Contribution is the percentage of times a variable is used to form branches in BRT models. PET = potential evapotranspiration. Tmin = minimum daily temperature.


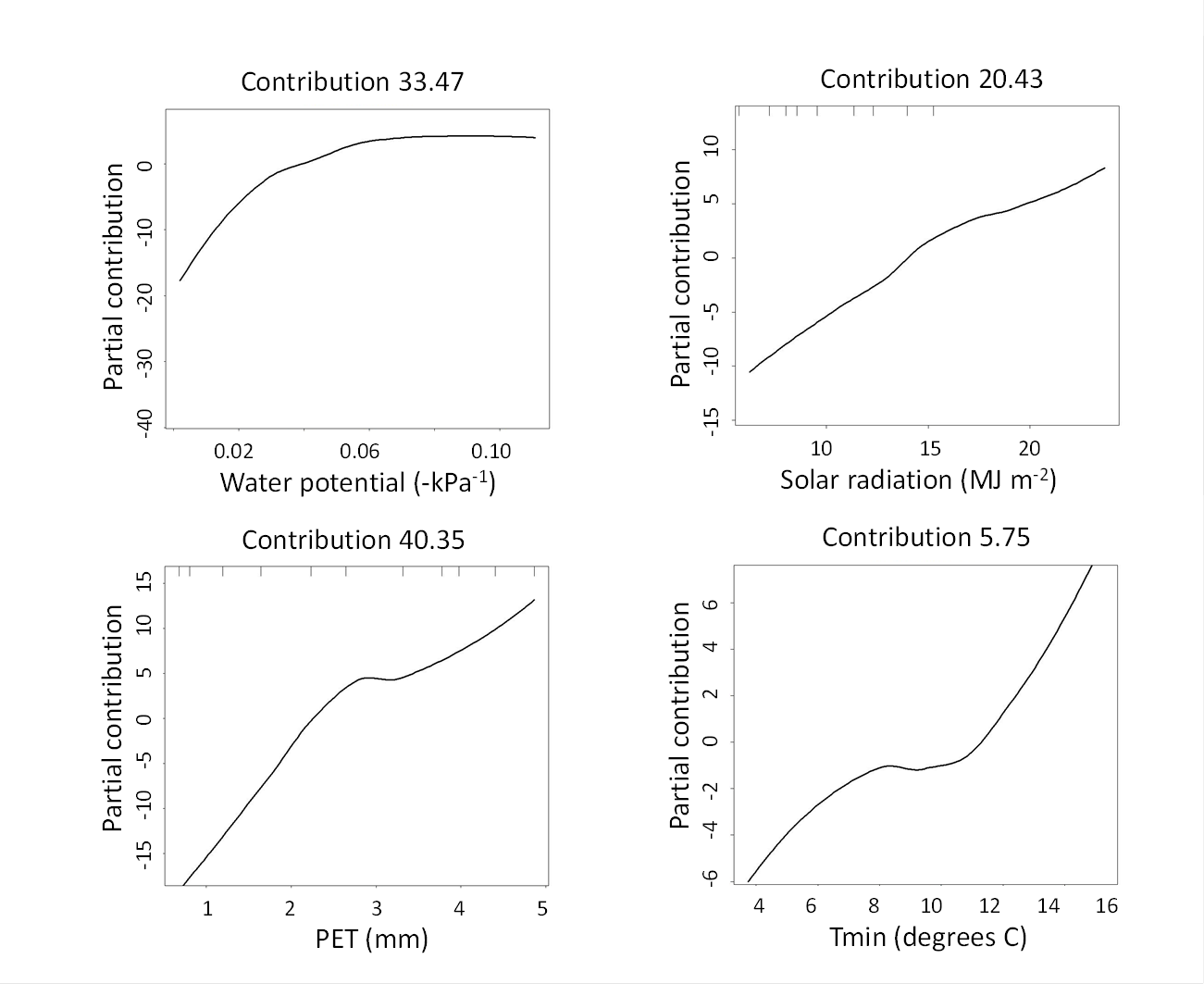


Figure S3: Partial response of predictors to productivity in the BRT model for the complex mixture (RGCO). Contribution is the percentage of times a variable is used to form branches in BRT models. PET = potential evapotranspiration. Tmin = minimum daily temperature.


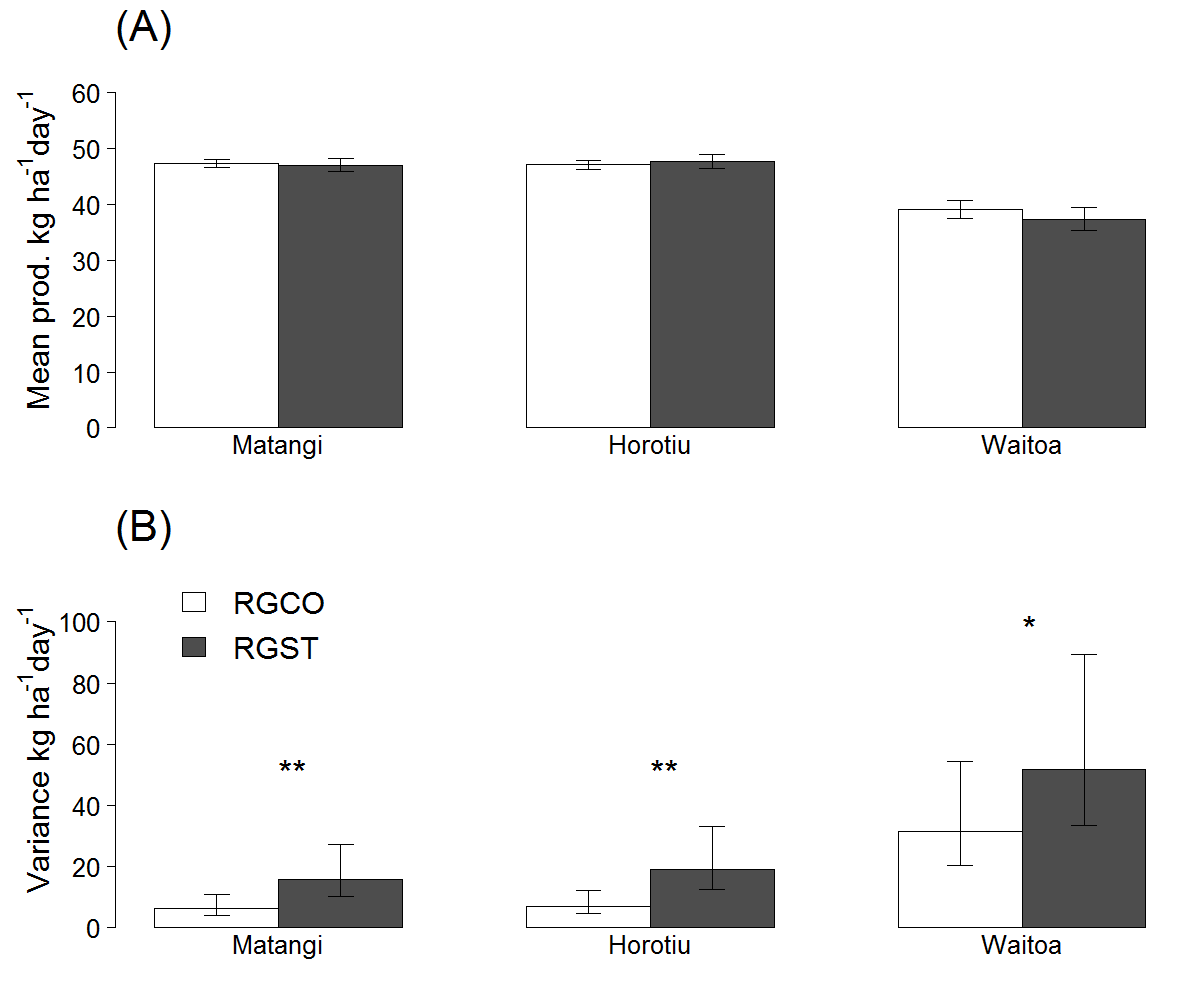


**Figure S4:** Long-term mean (A) and variance (B) of predicted annual productivity for the standard (RGST) and complex (RGCO) mixtures for each soil type occurring at Scott Farm. Error bars show 95% confidence intervals. * = *p* < 0.05; ** = *p* < 0.01. Predictions were produced using boosted regression tree (BRT) models fitted using both small and large plot harvest data and long-term climate data for the virtual climate station closest to the experimental site. See “predicting productivity USING long-term climate data for different soils at the field trial site” in the methods section for more detail.


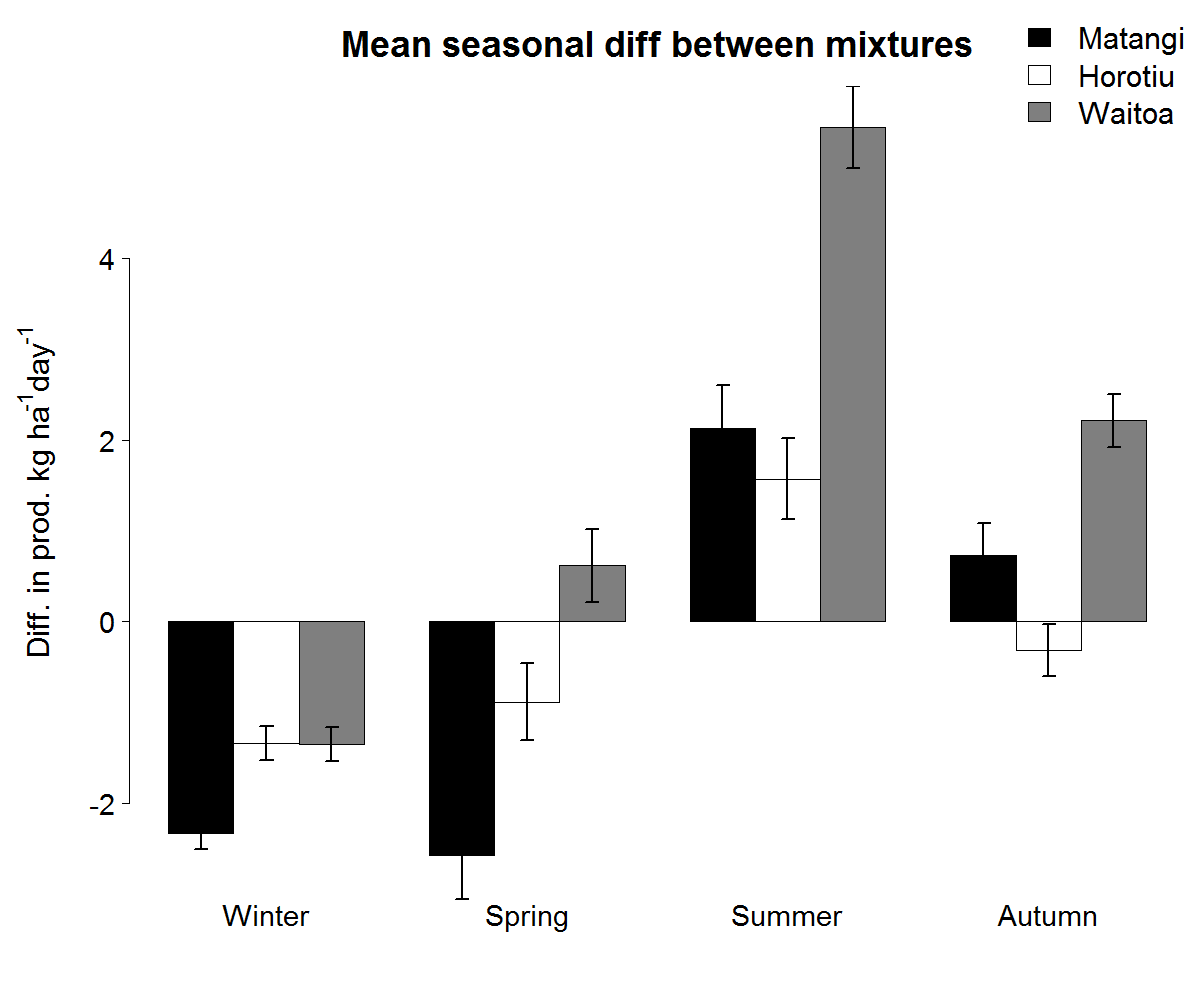


Figure S5: Differences in predicted long-term mean seasonal productivity between the standard and complex mixtures using climate data from the virtual climate station closest to the experimental site for the three soil types occurring at Scott Farm. Negative values indicate the standard mixture had greater productivity. Error bars show 95% confidence intervals. Predictions were produced using boosted regression tree (BRT) models fitted using both small and large plot harvest data and long-term climate data for the virtual climate station closest to the experimental site. See “predicting productivity USING long-term climate data for different soils at the field trial site” in the methods section for more detail.


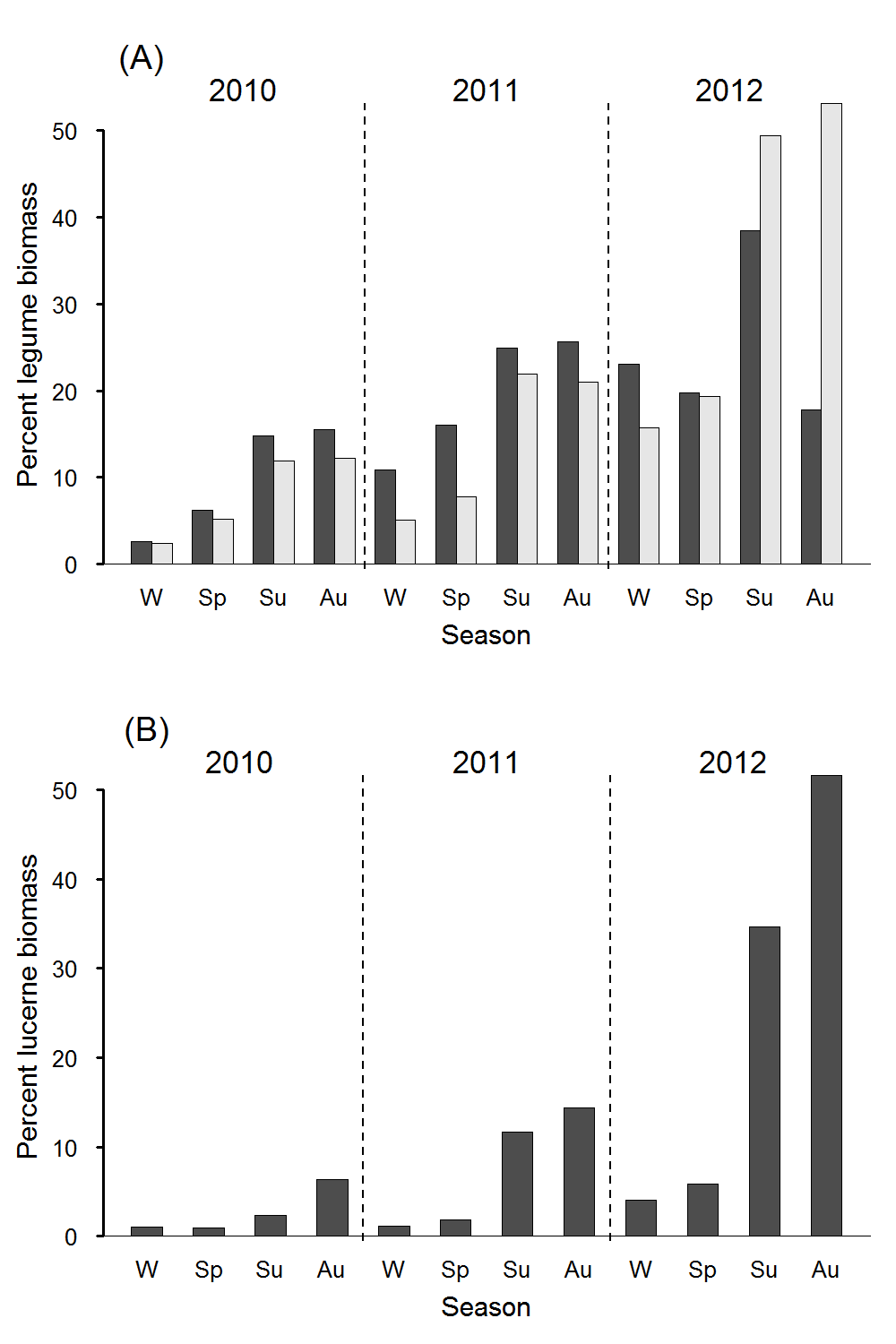


**Figure S6:** Percent of total biomass contributed by all legume species in each season for the standard (dark grey bars) and complex (light grey bars) mixtures (A) and by lucerne (*Medicago sativa*) in the complex mixture (B). These results are averaged across harvest data from both small and large plots. Season codes are: W , winter; Sp, spring; Su, summer; Au, autumn.
